# Role of ICAM-1 in triple-negative breast cancer

**DOI:** 10.1515/med-2024-0969

**Published:** 2024-05-21

**Authors:** Ying Zhang, Jingjing Fan, Xiaoli Wang, Zhongyu Wu, Weiqiang Ma, Binlin Ma

**Affiliations:** Xinjiang Medical University Affiliated Tumor Hospital, The Clinical Medical Research Center of Breast and Thyroid Tumor in Xinjiang, Urumqi, Xinjiang, 830000, China

**Keywords:** ICAM-1, the cancer genome atlas, biomarkers, immunotherapy, triple-negative breast cancer

## Abstract

Intercellular adhesion molecule-1 (ICAM-1) is related to the occurrence and development of a variety of tumors. However, the role of ICAM-1 in the regulation of growth, metastasis, and clinical prognosis of the specific molecular subtypes of breast cancer, triple-negative breast cancer (TNBC), remains to be elucidated. This study explored the role of ICAM-1 in breast cancer and its triple-negative subtypes by systematic bioinformatics methods. The results showed that the expression of ICAM-1 in breast cancer tissues was significantly higher than that in normal tissues, especially in TNBC subtypes. In breast cancer, ICAM-1 mainly activates pathways related to apoptosis and epithelial–mesenchymal transition, while its overexpression in TNBC is associated with inflammatory response, apoptosis, and other processes. TNBC patients displaying higher ICAM-1 expression demonstrate enhanced responses to immunotherapy. High ICAM-1 expression is sensitive to drugs targeting tumor cell proliferation, apoptosis, and angiogenesis. In conclusion, breast cancer is characterized by significantly high expression of ICAM-1, with TNBC subtypes expressing ICAM-1 at much higher levels than other subtypes. The diagnosis, prognosis, development, distant metastases, and immunotherapy of TNBC are correlated with high expression of ICAM-1. This research provides available data for the further study of the diagnosis and treatment of TNBC.

## 1 Introduction

With more new cases than lung cancer, breast cancer is now the most common cancer globally [1]. Approximately 15% of all occurrences of breast cancer are triple-negative breast cancer (TNBC), a unique subtype of the disease. It occurs mostly in young women and has a high degree of malignancy. In addition to a high incidence of distant metastasis and local recurrence, it is frequently accompanied by brain and visceral metastases [2,3]. Therefore, studying the expression levels of various molecules in TNBC to seek a breakthrough in clinical treatment has become a current research hotspot [4].

Intercellular adhesion molecule-1 (ICAM-1) glycoproteins on the cell surface are crucial for immune system operation, cell-to-cell transmission, and interactions with the extracellular matrix [5,6]. ICAM-1 has been shown in studies to give primary tumor cells an invasive phenotype, stimulate the initiation and growth of tumors, and serve as a prognostic indicator [7].

This study found that ICAM-1 was raised in several malignancies, including breast cancer, and considerably overexpressed in TNBC. Immunohistochemical studies revealed that TNBC cancer tissues expressed more ICAM-1 than paracancerous tissues; however, there are insufficient reports in the existing literature. Furthermore, this study investigated the association between ICAM-1 and immunological molecules, pathways, and infiltration of the immune system. It was discovered that this relationship was linked to drug sensitivity. Therefore, the purpose of this study is to examine the expression of ICAM-1 in malignancies, examine its involvement in the onset and progression of TNBC, and offer novel therapeutic treatment and diagnosis strategies utilizing its immunological function.

## 2 Materials and methods

### 2.1 Patients and databases

From the Cancer Genome Atlas Program (TCGA) database (https://portal.gdc.cancer.gov) built by NCI and the National Human Genome Research Institute to download and organize 33 kinds of tumor and TCGA - BRCA (breast ductal carcinoma,1226 cases by RNAseq,113 paracancerous samples) genome data and clinical data, analyzes the differences of expression and clinical significance. We divided the breast cancer patients with TCGA data into high and low expression groups according to the median expression of ICAM-1 and analyzed the association between the two groups and clinical characteristics. The data underwent receiver operating characteristic (ROC) analysis using the pROC tool, and ggplot2 was utilized to show the findings. Clinical data from BRCA patients in the TCGA were collected and assembled. Moreover, the precise expression level of ICAM-1 in various breast cancer subtypes was confirmed using two datasets from the Gene Expression Omnibus collection (GSE45827 and GSE36693).

Furthermore, 96 surgical specimens from patients with TNBC who had their initial diagnosis were gathered between January 2018 and April 2020. The tissue specimens were then divided into four groups: cancer (30 cases), paracarcinoma (30 cases), positive lymph node (20 cases), and negative lymph node (16 cases). The expression of ICAM-1 in different groups was detected. Inclusion criteria were as follows: (1) newly diagnosed triple negative breast cancer cases with HER2 (–), ER (–), and PR (–) confirmed by pathology and (2) patients and their family members agreed to retain tissue specimens for research. Exclusion criteria were as follows: (1) patients with other malignant tumors; (2) without preoperative anti-tumor treatment such as radiotherapy and chemotherapy; and (3) complicated with heart, liver, kidney, and other serious organ dysfunction or immune system diseases. All samples were embedded in paraffin, fixed in 10% formaldehyde solution, and sectioned at a thickness of 5 μm.

### 2.2 The expression of ICAM-1 in TNBC was discovered using immunohistochemistry

Surgical specimens collected in our hospital were used for further verification. The tissue slices were roasted at 65°C in an incubator for 1–2 h. Soak the tissue sections in xylene for 10 min and for another 10 min after replacing the xylene; in absolute alcohol Ⅰ and Ⅱ for 5 min; and in 95, 90, 80, and 70% alcohol and distilled water for 5 min, respectively. After boiling in a pressure cooker with 0.01 M citrate buffer (PH 6.0), the slices were placed in the repair solution, heated to exhaust, and the fire was turned off, so that the slices were immersed in the buffer for 10 min, and then, the lid was opened to cool at room temperature. The 3% H_2_O_2_ deionized water just formulated was used and immersed in it for 10 min to inactivate endogenous peroxidase. The stainless steel carrying basket was soaked in phosphate-buffered saline (PBS) and washed three times for 5 min each time. Take the appropriate proportion of diluted primary antibody (usually PBS, the exact dilution was determined by pre-experiment) and add 50 μL of anti-ⅰ antibody to each slide, overnight at 4℃ (the next day needs to be rewarmed at room temperature for 20 min). The stainless steel basket was soaked in PBS and washed three times for 5 min each time. 50 μL of horseradish peroxidase-labeled anti-Ⅱ was dropped and left for 20 min at room temperature. The stainless steel basket was soaked in PBS and washed three times for 3 min each time. After adding a drop of 50 μL of working streptavidin solution tagged with horseradish peroxidase, the mixture was allowed to settle for 20 min at room temperature. The stainless steel carrying basket was soaked in PBS and washed three times for 5 min each time. DAB color was developed for 3–10 min, and the degree of staining was mastered under the microscope (DAB color was brown granular precipitate). The color development was terminated for 3 min, followed by 3 min of hematoxylin counterstained, a few seconds of hydrochloric alcohol differentiation (1–2 s), and 5 min of reverse blue in tap water. The cells were submerged for 3 min each in distilled water and 70, 80, 90, and 95% alcohol and then for 5 min each in absolute ethanol I and II to dehydrate them. Xylene I and II were sealed after 5 min of transparency each and then baked at 37°C overnight.

All the staining results were double-blind read by two deputy chief pathologists of the department of pathology, and if there were inconsistent results, a unanimous conclusion was reached after discussion.

The evaluation criteria were immune response score (IRS), namely (1) according to the presence and depth of cell color: 0 points (no staining), 1 point (light yellow), 2 points (brown yellow), and 3 points (tan). (2) According to the percentage of chromogenic cells: 100 cells per high-power field were counted and scored as 0 points (cell positive rate <5%), 1 point (5–25%), 2 points (26–50%), and 3 points (cell positive rate >50%). The above scores were summed, 0 was defined as negative “1”, 1–2 was defined as weak positive “+”, 3–4 was defined as positive “++”, and 5–6 was defined as strong positive “+++”. The IRS value ≥4 was defined as high expression.

### 2.3 Breast cancer gene, pathway, and cellular functions identification in relation to ICAM-1

We used the ICAM-1 and other genetic correlation analyses, with a threshold for |cor| > 0.4 and a false discovery rate <0.05, to investigate the role of ICAM-1 in breast cancer biology. Correlation chordal plots show the genes that are highly related to ICAM-1. Cancer genome analysis platform GSCALite (http://bioinfo.life.hust.edu.cn/web/GSCALite/) is a single data processing pipeline for genomic analysis [8]; it combines medication response data from Genomics of Drug Sensitivity in Cancer (GDSC) and CTRP, normal tissue data from GTEx, and cancer genomic data from 33 cancer types from TCGA. On this platform, we investigated well-known cancer-associated pathways where ICAM-1 and its related genes are either activated or repressed in breast cancer. Furthermore, Gene Set Enrichment Analysis (GSEA) was used to carry out functional enrichment analysis of ICAM-1 in TNBC (*N* = 140) [9,10].

### 2.4 Immune infiltration analysis

The R [Rstudio software version 2023.06.1 + 524 (Posit Software Corporation, 2022, USA); R software version 4.2.1 (R Foundation for Statistical Computing, 2021, Austria)] package “ESTIMATE” was used to produce the ImmuneScore, which is correlated with the amount of immune cell infiltration, the StromalScore, which is correlated with the quantity of stromal cells, and the ESTIMATEScore, which is negatively correlated with tumor purity [11,12]. For immune infiltration analysis, the ImmuneScore, StromalScore, and ESTIMATEScore of the ICAM-1 gene were compared using the ESTIMATE algorithm. The R package “GSVA” was used to generate a single-sample genome enrichment analysis [13,14].

### 2.5 Drug susceptibility analysis

Target genes and related gene-drug susceptibility were explored by GSCALite. Using the “prophetic” R software package, which is a component of the GDSC effort, ridge regression modeling was used to determine the half-maximal inhibitory concentration (IC_50_) of several medicines in TCGA-BRCA. To evaluate the drug-susceptibility predictions, *t* tests were used to compare the IC_50_ values for specific drugs across risk groups.

### 2.6 Statistical treatment

The experimental data were assessed using SPSS software version 25.0 (IBM Corporation, 2017, USA), and the results were shown as mean ± standard deviation (±s). The two sets of data were compared using a rank sum test if the data did not follow a normal distribution; a test level of *α* = 0.05 and *p* < 0.05 (two-sided) were considered statistically significant. If the measurement data did not follow a normal distribution, an independent sample *t* test was used to compare them, and a two-test was used to compare the enumeration data.


**Informed consent:** Every participant in the study gave their informed consent.
**Ethical approval:** The study was approved by the Ethics Committee of Amend Letter of Ethics Committee of The Affiliated Cancer Hospital of Xinjiang Medical University (protocol code K-2022032 and date of March 4, 2022).

## 3 Results

### 3.1 ICAM-1 was abnormally upregulated in breast cancer

**Figure 1:**
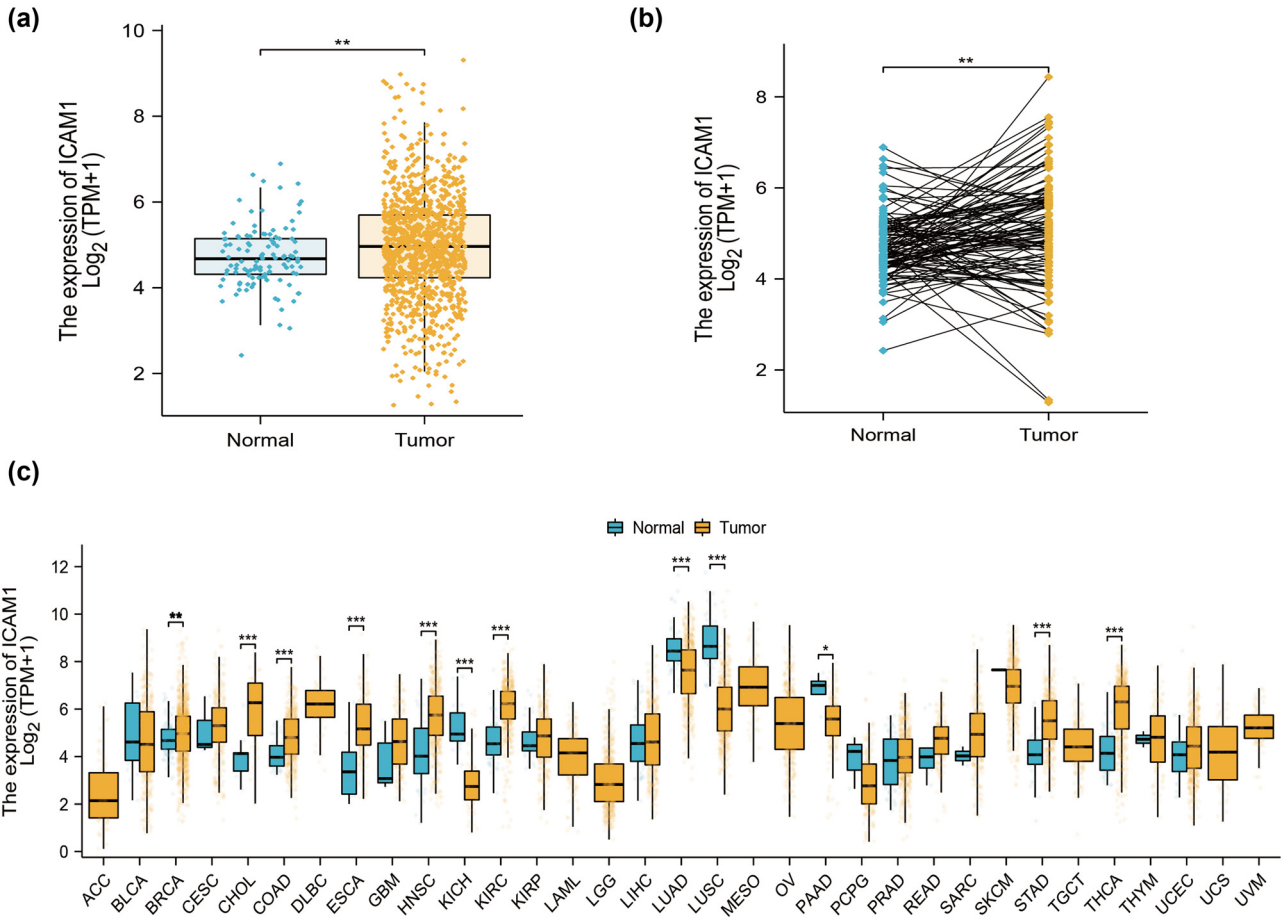
Differences in ICAM-1 expression in various cancers. (a) Expression of ICAM-1 mRNA in normal tissues and breast cancer. Breast cancer tissues showed significantly higher average expression levels of ICAM-1 mRNA as compared to normal tissues. (b) ICAM-1 expression in paired tumor and adjacent samples. (c) ICAM-1 expression levels in various cancers. ICAM-1 is overexpressed in many tumor types. *p < 0.05, **p < 0.01, ***p < 0.001.

The mRNA expression pattern of ICAM-1 in breast cancer tissues and normal tissues was discovered using TCGA databases. Compared to normal tissues, breast cancer tissues exhibited a considerably greater average expression level of ICAM-1 mRNA (*p* < 0.001, Figure 1a). Next, we examined ICAM-1 expression in neighboring samples and associated malignancies (Figure 1b). We performed a comprehensive analysis using data from the TCGA database to assess the expression level of ICAM-1 in various cancer types. Our study shows that ICAM-1 is overexpressed in eight different types of tumors: kidney renal clear cell carcinoma, breast invasive carcinoma, stomach adenocarcinoma, cholangiocarcinoma, esophageal carcinoma, head and neck squamous cell carcinoma, colon adenocarcinoma, and thyroid carcinoma; ICAM-1 expression was low in four different types of tumors: lung squamous cell carcinoma, kidney chromophobe, pancreatic adenocarcinoma, and lung adenocarcinoma; ICAM-1 expression was not different in the other 21 different types of tumors: bladder urothelial carcinoma, uterine carcinosarcoma, adrenocortical carcinoma, cervical squamous cell carcinoma and endocervical adenocarcinoma, uterine corpus endometrial carcinoma, skin cutaneous melanoma, lymphoid neoplasm diffuse large B-cell lymphoma, sarcoma, kidney renal papillary cell carcinoma, glioblastoma multiforme, prostate adenocarcinoma, acute myeloid leukemia, pheochromocytoma and paraganglioma, brain lower grade glioma, mesothelioma, liver hepatocellular carcinoma, rectum adenocarcinoma, ovarian serous cystadenocarcinoma, testicular germ cell tumors, uveal melanoma, and thymoma. Furthermore, Table 1 contains the clinical data of breast cancer patients that were obtained from TCGA. We found that the pathological stage, T stage, PAM50 classification, and PR, ER, and HER2 receptor status were also significantly correlated with the expression of ICAM-1 mRNA, and the *p* value was statistically significant.

**Table 1:** Relationship between BRCA patients’ clinicopathological characteristics and ICAM-1 expression

Characteristics	Low expression of ICAM-1	High expression of ICAM-1	*p* value	Method
*N*	543	544		
**Pathologic stage,** ***n*** **(%)**			0.015	Chisq test
Stage I	79 (7.4%)	103 (9.7%)		
Stage II	301 (28.3%)	318 (29.9%)		
Stage III	142 (13.4%)	102 (9.6%)		
Stage IV	8 (0.8%)	10 (0.9%)		
**Pathologic T stage,** ***n*** **(%)**			0.008	Chisq test
T1	121 (11.2%)	157 (14.5%)		
T2	315 (29.1%)	316 (29.2%)		
T3	85 (7.8%)	55 (5.1%)		
T4	20 (1.8%)	15 (1.4%)		
**Pathologic N stage,** ***n*** **(%)**			0.156	Chisq test
N0	244 (22.8%)	272 (25.5%)		
N1	180 (16.9%)	179 (16.8%)		
N2	56 (5.2%)	60 (5.6%)		
N3	47 (4.4%)	30 (2.8%)		
**Pathologic M stage,** ***n*** **(%)**			0.662	Chisq test
M0	452 (48.9%)	453 (49%)		
M1	9 (1%)	11 (1.2%)		
**Age,** ***n*** **(%)**			0.260	Chisq test
≤60	292 (26.9%)	311 (28.6%)		
>60	251 (23.1%)	233 (21.4%)		
**Histological type,** ***n*** **(%)**			0.080	Yates’ correction
Infiltrating ductal carcinoma	376 (37.1%)	400 (39.4%)		
Infiltrating carcinoma NOS	0 (0%)	1 (0.1%)		
Medullary carcinoma	1 (0.1%)	5 (0.5%)		
Metaplastic carcinoma	7 (0.7%)	2 (0.2%)		
Mucinous carcinoma	12 (1.2%)	5 (0.5%)		
Infiltrating lobular carcinoma	103 (10.2%)	102 (10.1%)		
**OS event,** ***n*** **(%)**			0.158	Chisq test
Alive	459 (42.2%)	476 (43.8%)		
Dead	84 (7.7%)	68 (6.3%)		
DSS event, *n* (%)			0.088	Chisq test
No	483 (45.3%)	499 (46.8%)		
Yes	50 (4.7%)	35 (3.3%)		
**PAM50,** ***n*** **(%)**			＜0.001	Chisq test
Normal	15 (1.4%)	25 (2.3%)		
LumA	302 (27.8%)	262 (24.1%)		
LumB	129 (11.9%)	77 (7.1%)		
HER2	38 (3.5%)	44 (4%)		
Basal	59 (5.4%)	136 (12.5%)		
**PR status,** ***n*** **(%)**			0.001	Chisq test
Negative	145 (14%)	197 (19.1%)		
Positive	367 (35.5%)	325 (31.4%)		
**ER status,** ***n*** **(%)**			＜0.001	Chisq test
Negative	85 (8.2%)	155 (14.9%)		
Positive	428 (41.3%)	369 (35.6%)		
**HER2 status,** ***n*** **(%)**			0.096	Chisq test
Negative	254 (35.4%)	306 (42.7%)		
Positive	83 (11.6%)	74 (10.3%)		

Note: OS: overall survival, DSS: disease-specific survival.

### 3.2 For TNBC, ICAM-1 can be utilized as a particular diagnostic and prognostic marker

**Figure 2:**
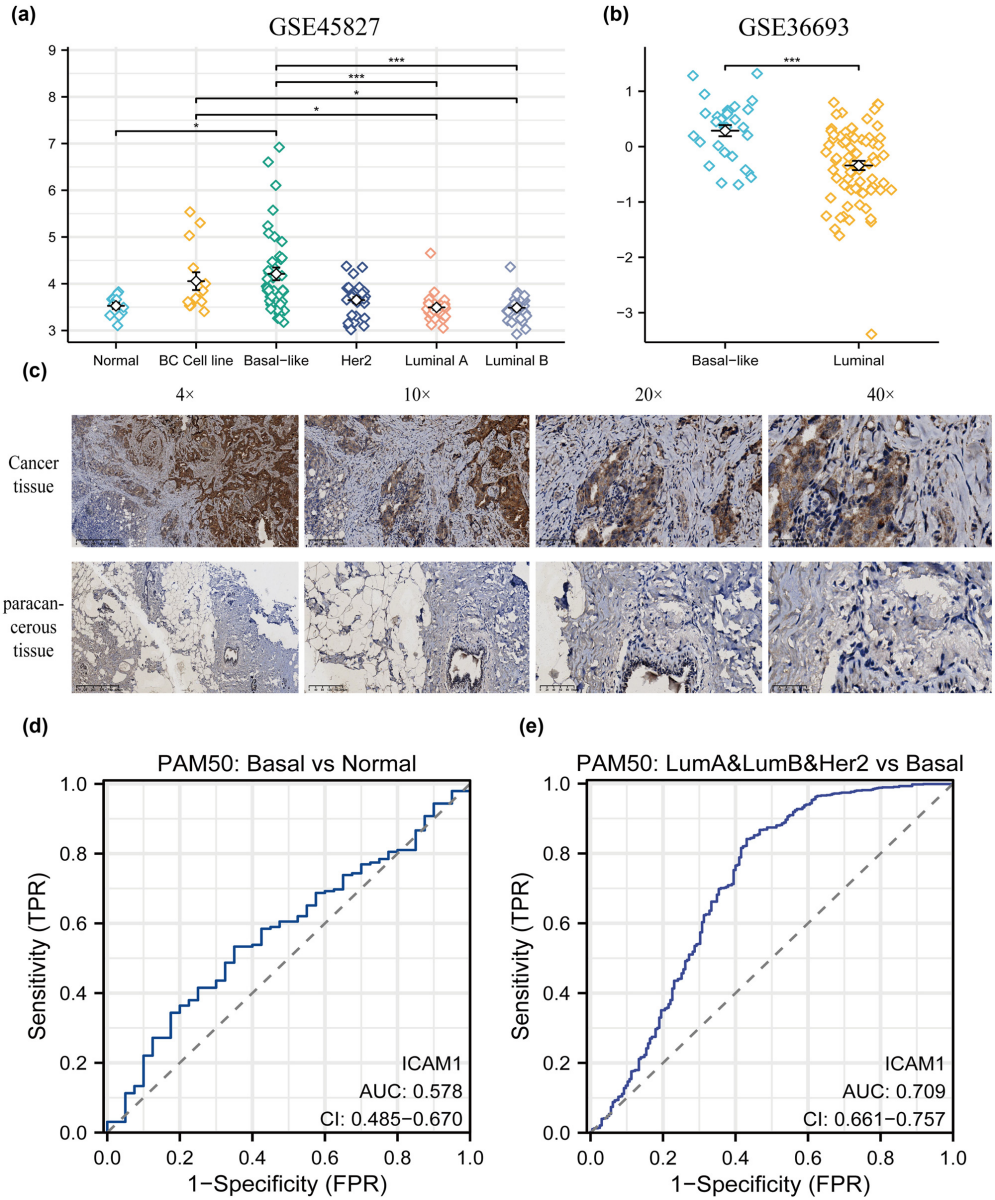
Expression and clinical significance of ICAM-1 in TNBC. (a and b) Expression of ICAM-1 in different breast cancer types in GSE45827 and GSE36693 datasets. (BC cell line: 1 Cell Luminal BT474; 1 Cell Luminal MB361; 5 Cell Luminal MB415; 1 Cell Luminal MCF7; 1 Cell Luminal SKBR3; 1 Cell Luminal T47D). ICAM-1 was highly expressed in TNBC subtypes. (c) Clinical sample immunohistochemical plot of paracancerous and cancerous tissue. TNBC cancer tissues expressed more ICAM-1 than paracancerous tissues did. (d) ROC curve of ICAM-1 in breast cancer cohort; the AUC was 57.8%. (e) ROC curve of ICAM-1 in other subtypes of breast cancer and TNBC; the AUC was 70.9%. *p < 0.05, **p < 0.01, ***p < 0.001.

We surmise that ICAM-1 may play a significant role in TNBC based on the clinicopathological features of the patients, where the high expression of ICAM-1 is associated with PR^−^-, ER^−^-, and HER2^−^-positive cells. The assessment of ICAM-1 expression in different subtypes of breast cancer was thus conducted by validation utilizing two datasets: GSE45827 and GSE36693. The results showed that ICAM-1 expression in TNBC was, in fact, much higher than in other subtypes (Figure 2a and b).

We further verified the above results using surgical specimens from our hospital. Immunohistochemical micrographs are shown in Figure 2c. Immunohistochemical results showed that: the expression of ICAM-1 was 4.767 ± 1.278 in triple-negative breast cancer tissues, 2.700 ± 2.548 in paracancerous tissues, and 3.800 ± 1.105 in positive lymph nodes. The expression of ICAM-1 in the negative lymph node group was 3.000 ± 1.211 (the differences in indicator expression between the cancer group and the surrounding tissues, as well as between the positive and negative lymph node groups, were verified in this section using the rank sum test).TNBC cancer tissues expressed more ICAM-1 than the surrounding tissues did (*p* < 0.05) (Table 2). Furthermore, the TCGA data set’s ROC curve analysis was utilized to thoroughly assess the sensitivity and specificity of ICAM-1 for TNBC and breast cancer. In the breast cancer cohort, the ICAM-1 area under the curve (AUC) was 57.8% (Figure 2d). In comparison to other breast cancer subtypes, the ICAM-1 AUC was 70.9% in TNBC (Figure 2e). Therefore, we believe that ICAM-1 may be a specific marker for TNBC.

**Table 2:** Differences in the distribution of immunohistochemical scores of ICAM-1 indicators in different populations

Grouping	Number of cases	ICAM-1 indicator score							*p* values
		0	1	2	3	4	5	6	
Cancer group	30	0 (0%)	0 (%)	2 (7%)	4 (13%)	4 (13%)	9 (30%)	11 (37%)	<0.001
Paracancerous group	30	12 (40%)	1 (3%)	2 (7%)	0 (0%)	6 (20%)	2 (7%)	7 (23%)	
Positive lymph node group	20	0 (0%)	0 (0%)	4 (20%)	1 (5%)	11 (55%)	3 (15%)	1 (5%)	0.392
Negative lymph node group	16	1 (6%)	0 (0%)	5 (31%)	2 (13%)	8 (50%)	0 (0%)	0 (0%)	

Note: The data in the table are the number of people in each population for each score, and the proportion of the number in each population is in parentheses.

### 3.3 Identification of ICAM-1-related pathways, genes, and cell functions in breast cancer

**Figure 3:**
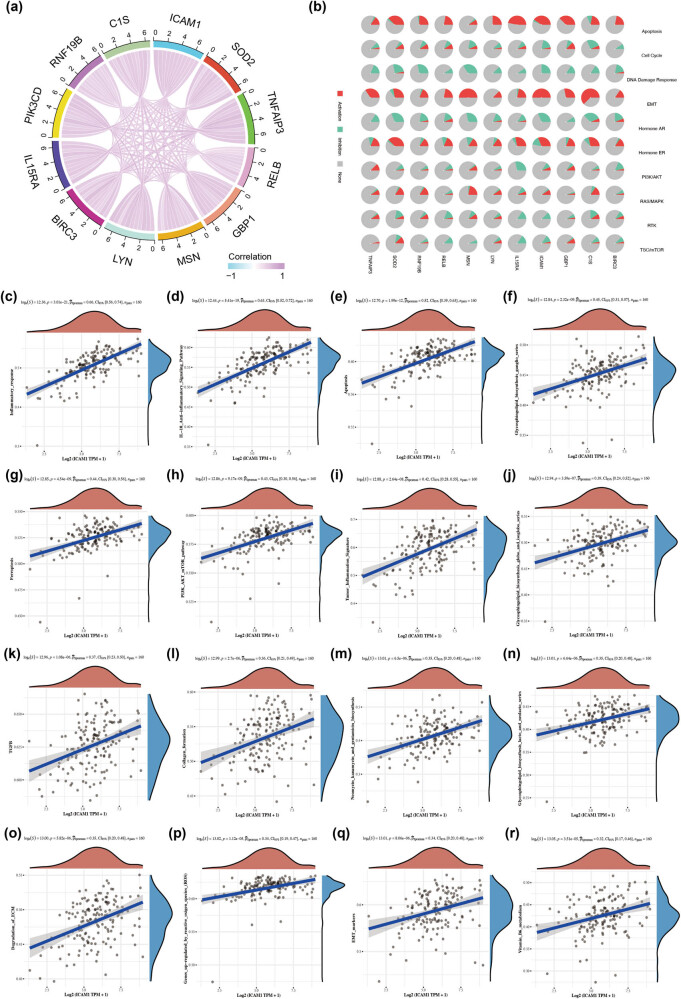
Functional enrichment analysis. (a) SOD2, TNFAIP3, RELB, GBP1, MSN, LYN, BIRC3, IL15RA, PIK3CD, RNF19B, C1S, etc. The above genes are highly associated with ICAM-1. (b) Mechanisms linked to cancer that regulate the expression of ICAM-1 and associated genes in breast cancer; Spearman correlation analysis between (c)–(r): ICAM-1 expression and pathway scores. Gene expression is represented by the *X*-axis, pathway score by the *Y*-axis, pathway score distribution trend by the density curve on the right, gene expression distribution trend by the upper density curve, and correlation *p* value, correlation coefficient, and correlation calculation method by the upper value (blue curve in the coordinate axis).).

Based on RNA sequencing data from the TCGA database, co-expression analysis of ICAM-1-related genes was carried out, and Figure 3a displays the interaction between 11 genes (SOD2, TNFAIP3, RELB, GBP1, MSN, LYN, BIRC3, IL15RA, PIK3CD, RNF19B, C1S, etc.) that have a strong correlation with ICAM-1. The well-known pathways in which ICAM-1 and associated genes are either activated or repressed in breast cancer are depicted in Figure 3b. The activated pathways of ICAM-1 were mainly apoptosis and epithelial–mesenchymal transition (EMT), and the inhibited pathways were mainly cell cycle, DNA damage response, AR hormones, and RTK signaling pathways. The results of the GSEA revealed that TNBC is associated with the following: inflammatory response and overexpression of ICAM-1; IL-10 anti-inflammatory signaling pathway; apoptosis; ganglionic glycosphingoliposis synthesis pathway; ferroptosis; PI3K-AKT-mTOR signaling pathway; tumor inflammatory characteristics; globo/iso-globo glycosphingoliposis synthesis pathway; transforming growth factor-β (TGF-β) signaling pathways; collagen formation; neomycin/kanamycin/gentamicin biosynthesis; lacto/neo-lacto sphingolipid synthesis pathway; extracellular matrix degradation; genes related to reactive oxygen species-related up-regulated genes; EMT markers; and vitamin B6 metabolism (Figure 3c–r). These findings imply that TNBC development, inflammation, metabolism, and apoptosis may be correlated with ICAM-1. On the other hand, ICAM-1’s function in tumor inflammatory response and metabolism emerged after breast cancer was reclassified as TNBC.

### 3.4 TNBC patients with high ICAM-1 expression have better immunotherapy response

**Figure 4:**
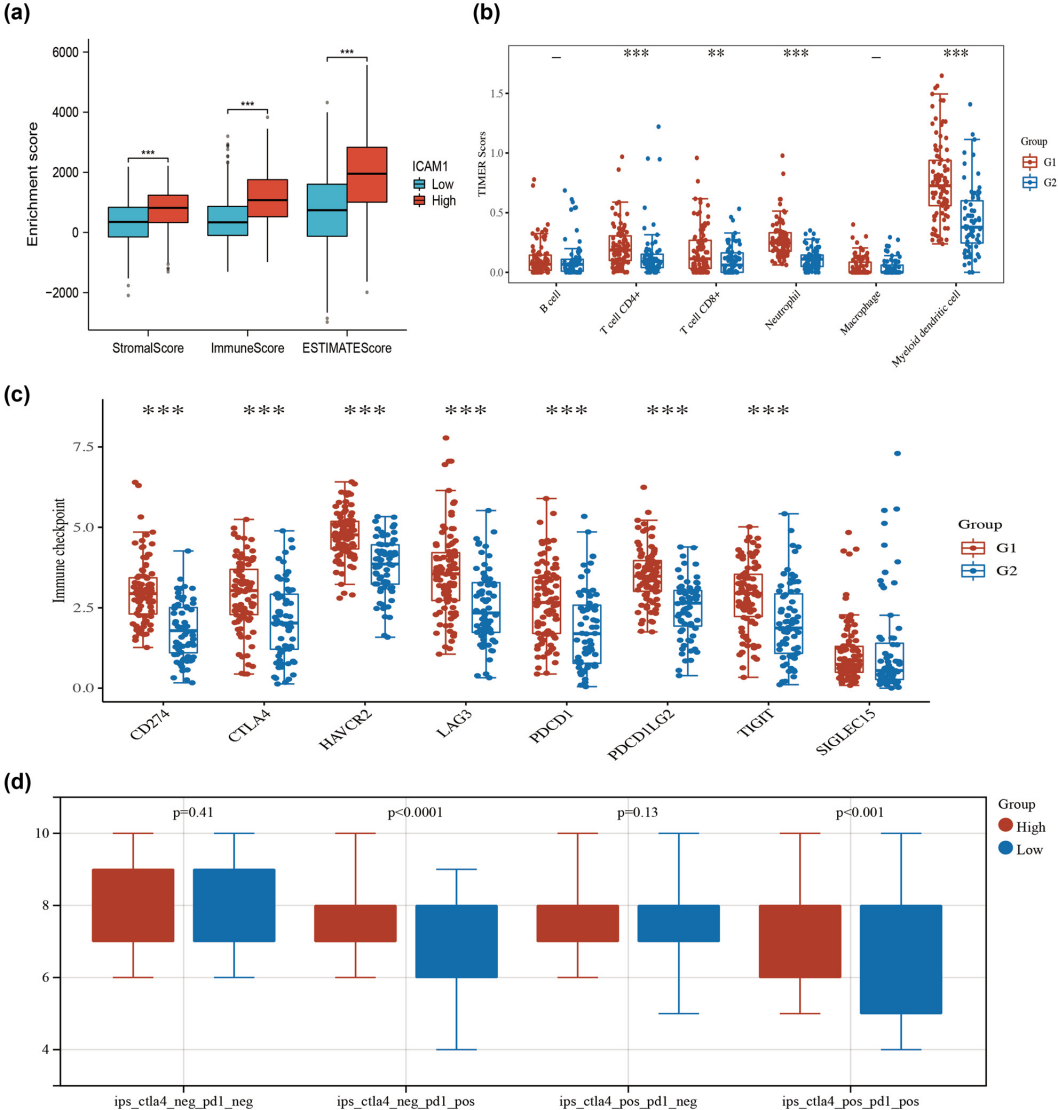
TNBC patients with high ICAM-1 expression have better immunotherapy response. (a) The percentage of immune and stromal cells in the tumor environment, as well as the stromal, immunological, and estimate scores of the ICAM-1 high expression group, were all noticeably higher than those of the low-risk group. (b) Differences in tumor microenvironment between ICAM-1 high expression group and low expression group in TNBC. (c) The variations in immunological checkpoints between the TNBC groups with high and low expression of ICAM-1. High expression levels of CD274, HAVCR2, CTLA4, PDCD1, LAG3, TIGIT, and PDCD1LG2 were seen in the high-risk group. (d) The sensitivity of immunotherapy in different ICAM-1 expression groups in TNBC. The high expression group’s IPS of CTLA4−/PD1+ and CTLA4+/PD1+ were considerably greater than those of the low expression group. *p < 0.05, **p < 0.01, ***p < 0.001.

We used the ESTIMATE program for tumor microenvironment score to calculate the proportion of immune and stromal cells in the tumor environment. The findings demonstrated that the ICAM-1 high expression group’s breast cancer tissues had greater stromal, immunological, and overall ESTIMATE scores than the low expression group’s, indicating a considerably higher rate of immune cell infiltration in the high expression group (Figure 4a). Further analysis was done on the variations in the tumor microenvironment between the high expression group and the low expression group in TNBC. TIMER was used to screen six tumor-infiltrating immune cells in total (Figure 4b). We also examined the variations in immunological checkpoints between the high-expression group and the low-expression group (Figure 4c). The findings indicated that the high-risk group had higher levels of expression for CD274, PDCD1LG2, HAVCR2, CTLA4, PDCD1, LAG3, and TIGIT. We compared the sensitivity to immunotherapy by various ICAM-1 expression groups in TNBC, assessing the response to immune checkpoint inhibitor treatment by immunohistochemical positive score (IPS). Higher IPS scores predicted a better response to immune checkpoint inhibitors treatment (divided into four classes, including PD-1 inhibitor and CTLA4 inhibitor treatment) (Figure 4d). The high expression group’s CTLA4−/PD1+ and CTLA4+/PD1+ IPS were both significantly higher than the low expression group’s, suggesting that the high expression group’s anti-PD-1 treatment and combined anti-PD-1 and anti-CTLA4 treatment were superior to the low expression group’s. These results suggest a substantial correlation between immune cell infiltration and immunotherapy in TNBC, as well as an improvement in immunotherapy responses in patients expressing high levels of ICAM-1.

### 3.5 Drug sensitivity analysis of ICAM-1 and related genes

**Figure 5:**
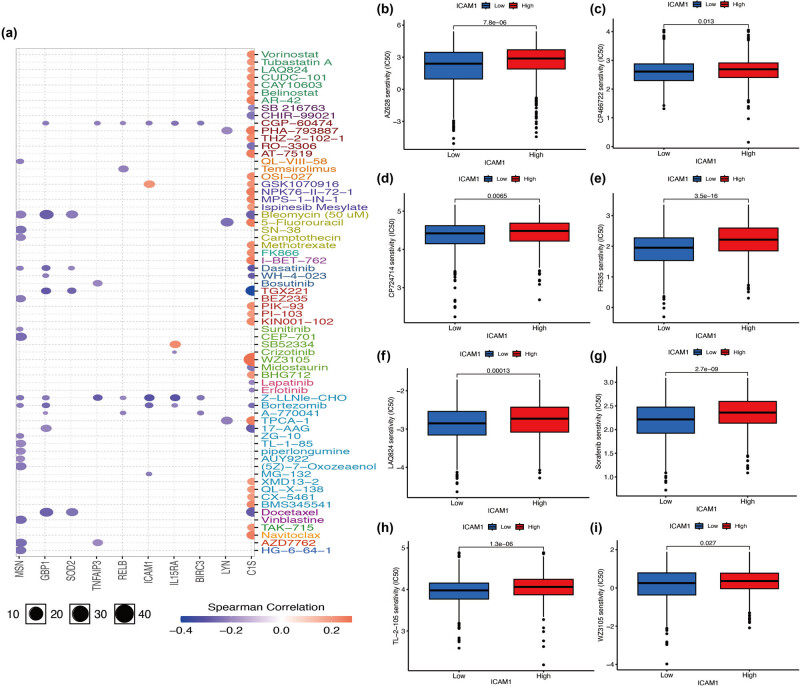
Analyzing the drug sensitivity of ICAM-1 and associated genes. (a) Drug sensitivity of ICAM-1 and related genes in the GSCALite database. The results showed that ICAM-1-related drugs were GSK1070916. (b–i) In the GSCALite database, there is a correlation between ICAM-1 expression and drug sensitivity. According to the findings, AZ628, CP466722, CP724714, FH535, LAQ824, sorafenib, TL-2-105, and WZ3015 were all effective at reducing ICAM-1 high expression.

Figure 5a shows the drug sensitivity of the target gene ICAM-1 and related genes in the GSCALite database. GSK1070916 was found to be related to ICAM-1. Figure 5b–i illustrates the relationship between ICAM-1 expression and drug sensitivity. Sensitivity to AZ628, CP466722, CP724714, FH535, LAQ824, sorafenib, TL-2-105, and WZ3105 was shown with high expression of ICAM-1. CP466722 has the ability to impede ATM-dependent phosphorylation in MCF7 breast cancer cells, thereby impeding the growth of tumor cells. CP-724714 has the ability to cause tumor cells to undergo apoptosis and induce G1 phase arrest in breast cancer cells. LAQ824 is a new and potent histone deacetylase inhibitor that has the ability to cause human breast cancer cells SKBR-3, BT-474, and MB-468 to undergo apoptosis.

## 4 Discussion

Breast cancer, one of the most common tumors in women, is on the rise. However, as novel approaches to treatment have gained acceptance, the death rate of breast cancer has gradually declined globally, making it one of the most successful solid tumors [15]. At present, breast cancer immunotherapy has been gradually explored and applied to clinical treatment [16]. The modulation of the autoimmune system is crucial to the therapy and long-term survival of triple negative breast cancer, a rare kind of breast cancer that has few treatment options [17]. Based on the above reasons, the research on the specific molecules of triple negative breast cancer is very important, and the discovery and exploration of various molecular markers has brought new hope to patients. The involvement of ICAM-1 in triple negative breast cancer is the primary topic of this article. CD54, or ICAM-1, is a transmembrane glycoprotein that is produced by immunological and endothelial cells [18]. This significant adhesion molecule plays a role in immune response, tumor degeneration, and metastasis [19]. Research indicates that ICAM-1 is present in exosomes produced from tumors and contributes to the contact between exosomes and T cells, which is necessary for immunological suppression mediated by PD-L1 [20]. When in touch with T cells, ICAM-1 can change the growth and development of tumor cells. This means that it may be useful in the immunotherapy of TNBC when normal chemotherapy and molecular targeted therapy are ineffective [21,22]. In addition, relevant literature suggests that ICAM-1 can promote metastasis by activating cellular pathways related to cell cycle and stem cells in lung metastasis of TNBC [23]. ICAM-1 increases cell invasiveness in TNBC bone metastases by inducing the EMT via TGF-β/SMAD signaling [24]. Given that ICAM-1 is involved in the disease’s distant metastasis, these studies suggest that it could be a new treatment target for TNBC metastasis.

Our initial analysis of ICAM-1 expression in many cancers using the TCGA database revealed that it was substantially expressed in a range of malignancies, including breast cancer. Based on these facts, the association between TNBC and breast cancer subtypes was examined in light of the distinct molecular subtypes of the disease and their various attributes [25]. We hypothesized that ICAM-1 may be a specific marker for TNBC based on the analysis results, which demonstrated that ICAM-1 was significantly highly expressed in the basal-like breast cancer subtypes and that ICAM-1 expression was highest in the PR-negative group and ER-negative group. As a result, we believe that ICAM-1 may be highly correlated with TNBC. To investigate ICAM-1 expression in breast cancer further, we obtained tissue samples from TNBC patients and categorized them for immunohistochemistry. In line with database prediction, the results demonstrated that TNBC tissues had higher levels of ICAM-1 expression than paracancerous tissues. to learn more about ICAM-1. Its importance in diagnostics, associated mechanisms, and treatment resistance are also covered in this article. ICAM-1 is linked to several pathways, such as IL2/STAT5, IL6/JAK/STAT3, and others and can assist in differentiating between luminal type and TNBC. Consequently, TNBC occurrence and development, inflammatory response, metabolism, and apoptosis may all be correlated with ICAM-1. On the other hand, ICAM-1’s function in tumor inflammatory response and metabolism emerged after breast cancer was reclassified as TNBC. Regarding drug resistance, this study discovered that the group exhibiting high ICAM-1 expression can be susceptible to eight medications, such as sorafenib, which are linked to angiogenesis, apoptosis, and tumor cell proliferation. Among these, CP466722, CP-724714, and LAQ824 have the ability to stop breast cancer cells from proliferating and induce the death of tumor cells. Regarding prognosis, it has been reported that ICAM-1 participates in leukocyte adhesion, motility, and immunological activation in tumor immunity [26], and immunotherapy may enhance the prognosis of patients at high risk. Currently, therapy approaches connected to ICAM-1 are also being investigated. For example, ICAM-1 can target near-infrared light immunotherapy (NIR-PIT) to inhibit tumor growth and improve the survival rate of TNBC patients [27,28]. ICAM-1 can mediate the interpore drug delivery system or target certain special nanoparticles to treat TNBC [29,30], etc. The interaction mechanism between ICAM-1 and different molecules and pathways remains unclear, the research on ICAM-1’s impact on the prognosis of breast cancer is incomplete, and the role of ICAM-1 in the diagnosis and treatment of breast cancer has not yet been fully realized. These are some of the limitations of the current ICAM-1 research. The findings of this work offer a foundation for the aberrant expression of ICAM-1 and its associated roles in TNBC subtypes of breast cancer; nevertheless, additional experimental validation is required to determine the precise function and intrinsic mechanism of this protein. Furthermore, this study’s comprehensive examination of ICAM-1 revealed that the protein is linked to several signaling pathways that are connected to TNBC and medications related to tumors, opening the door to immunotherapy and targeted therapy for TNBC. However, this portion of the investigation still requires experimental validation.

In summary, ICAM-1 is differentially expressed in a variety of tumors, with a notable upregulation in breast cancer, particularly triple negative breast cancer. In different types of breast cancer, ICAM-1 is highly correlated with TNBC, which can promote the occurrence and development of breast cancer and distant metastasis, and may be related to prognosis, immunotherapy, and drug resistance. Therefore, ICAM-1 plays an important role in tumor immunity, and its in-depth study may be a new hope for TNBC patients.
